# Current Perspective: 3D Spheroid Models Utilizing Human-Based Cells for Investigating Metabolism-Dependent Drug-Induced Liver Injury

**DOI:** 10.3389/fmedt.2020.611913

**Published:** 2020-11-30

**Authors:** Christopher R. Cox, Stephen Lynch, Christopher Goldring, Parveen Sharma

**Affiliations:** ^1^Department of Pharmacology and Experimental Therapeutics, MRC Centre for Drug Safety Science, Institute of Systems, Molecular & Integrative Biology, University of Liverpool, Liverpool, United Kingdom; ^2^Department of Cardiovascular and Metabolic Medicine, Institute of Life Course and Medical Sciences, University of Liverpool, Liverpool, United Kingdom; ^3^Liverpool Centre for Cardiovascular Science, Liverpool, United Kingdom

**Keywords:** 3D, spheroids, liver, hepatocyte, HLC, DILI, metabolism, *in-vitro*

## Abstract

Drug-induced liver injury (DILI) remains a leading cause for the withdrawal of approved drugs. This has significant financial implications for pharmaceutical companies, places increasing strain on global health services, and causes harm to patients. For these reasons, it is essential that *in-vitro* liver models are capable of detecting DILI-positive compounds and their underlying mechanisms, prior to their approval and administration to patients or volunteers in clinical trials. Metabolism-dependent DILI is an important mechanism of drug-induced toxicity, which often involves the CYP450 family of enzymes, and is associated with the production of a chemically reactive metabolite and/or inefficient removal and accumulation of potentially toxic compounds. Unfortunately, many of the traditional *in-vitro* liver models fall short of their *in-vivo* counterparts, failing to recapitulate the mature hepatocyte phenotype, becoming metabolically incompetent, and lacking the longevity to investigate and detect metabolism-dependent DILI and those associated with chronic and repeat dosing regimens. Nevertheless, evidence is gathering to indicate that growing cells in 3D formats can increase the complexity of these models, promoting a more mature-hepatocyte phenotype and increasing their longevity, *in vitro*. This review will discuss the use of 3D *in vitro* models, namely spheroids, organoids, and perfusion-based systems to establish suitable liver models to investigate metabolism-dependent DILI.

## Introduction

Drug-induced liver injury (DILI) is associated with a large proportion of withdrawn drugs (1) and is a leading cause of acute liver failure (2). This puts considerable strain on global health services and can have significant financial implications for pharmaceutical companies. Understanding the mechanisms that underlie DILI, therefore, remains an ongoing challenge in pharmacology and toxicology research. DILI may be associated with immunological reactions, diet-drug or drug-drug interactions, genetic variation in drug metabolizing enzymes and transporters (DMETs), or any combination of these factors (3, 4). This makes them extremely difficult to investigate, and, unfortunately, there is no perfect model system to study DILI, with different models varying in their complexity, relevance, cost, and ethical acceptance. Although *in-vivo* animal studies are necessary for getting a drug's approval, their use is a contentious area and the translatability of animal data to humans can also be low (5, 6). Hence, developing new *in-vitro* models that utilize human-derived cells provides a more ethically acceptable alternative that may be more translatable. Although there have been a number of comprehensive reviews on the different *in-vitro* liver models that can be used to evaluate a drug's toxicity and study DILI (7–12), this review will discuss the use of human-based cells to establish *in-vitro* models to study metabolism-dependent DILI, including the metabolic capabilities of 3D cultures, co-cultures, and perfusion-based platforms.

## The Role of the Liver

**Figure 1:**
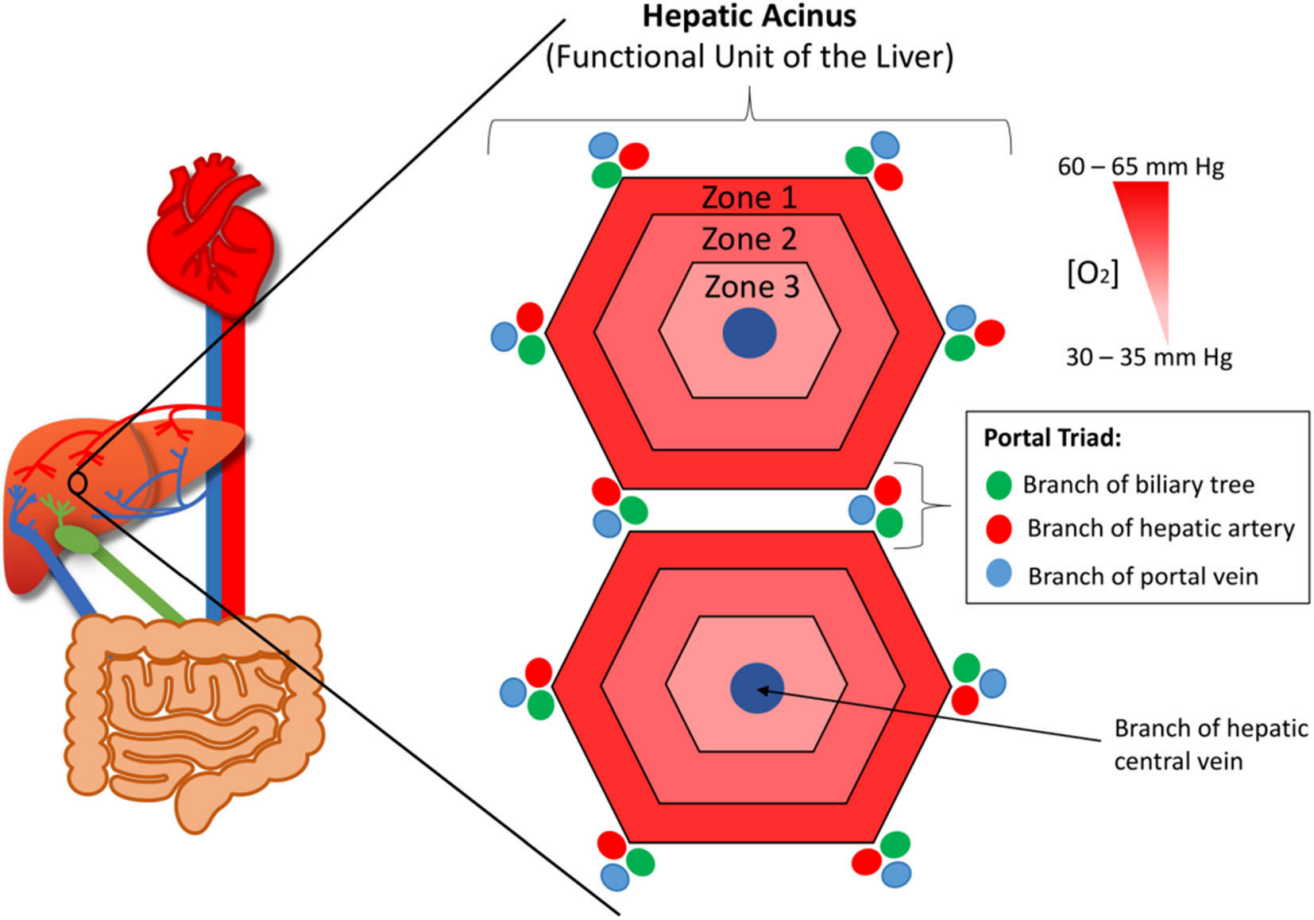
The functional unit or lobule of the liver (aka. The hepatic acinus). At each corner of each lobule is a portal triad, which contains branches of the hepatic portal vein, hepatic artery, and biliary tree. At the center of each lobule is the centrilobular vein. The lobule has an oxygen concentration gradient across it, with zone 1 having the highest concentration and zone 3 the lowest.

**Figure 2:**
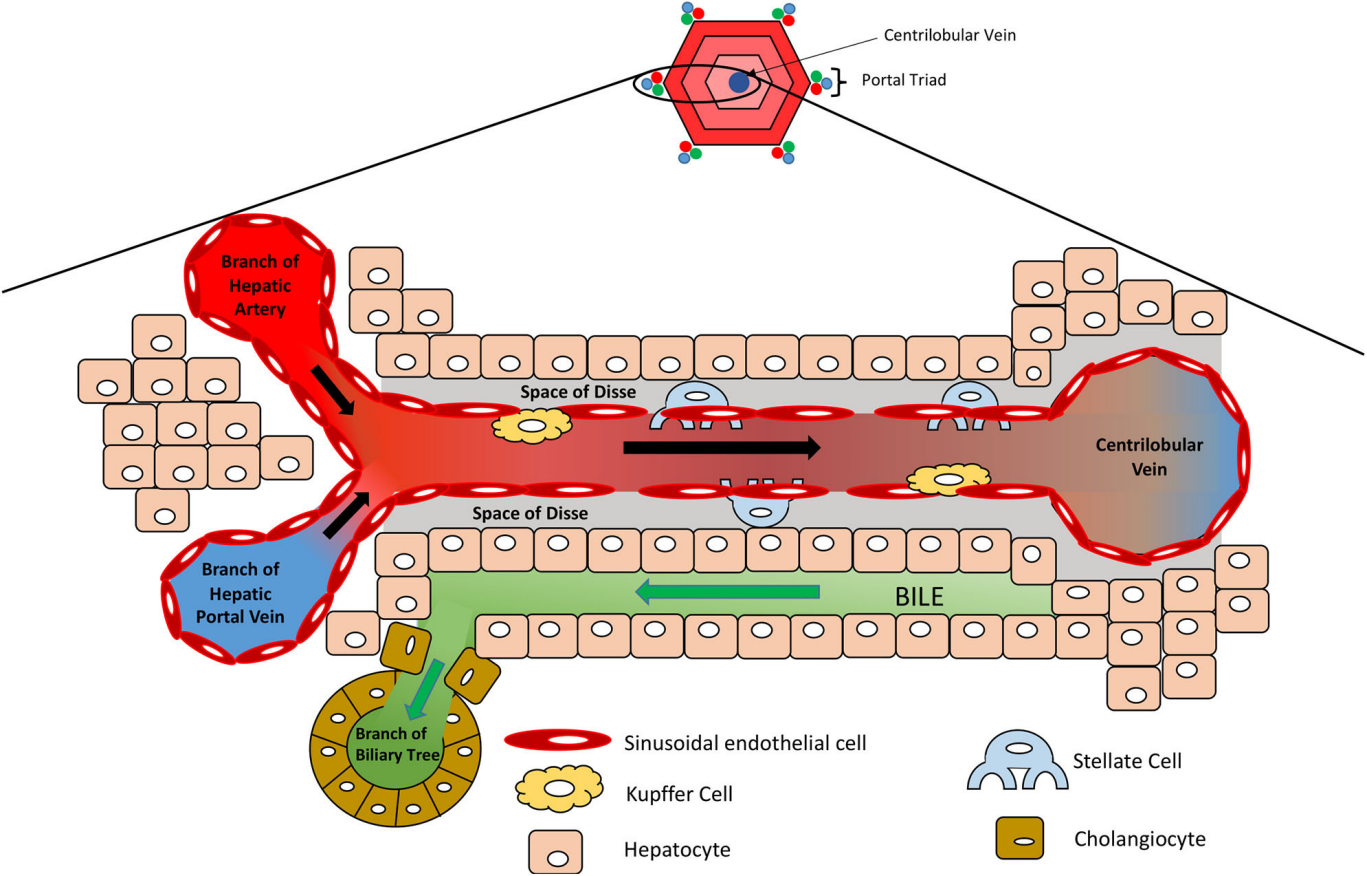
Microanatomy of the hepatic lobule. Oxygenated blood and nutrient-rich blood enter the sinusoid from the hepatic artery and portal vein, respectively, which flows to the center of the lobule where it enters the systemic circulation via the centrilobular vein. Sinusoidal endothelial cells line the sinusoid, which is surrounded by hepatocytes. Kupffer cells reside along the sinusoidal wall, removing any foreign particles, whilst stellate cells are localized in the space of Disse and act as storage cells for vitamin A.

The liver provides the primary route for compounds to enter the systemic circulation from the gastrointestinal tract. It has high metabolic capabilities to metabolize and modify these incoming compounds, a process known as first pass metabolism, and provides a major route for elimination via bile canaliculi and the bile ducts. The human liver consists of two lobes that contain functional units or lobules called “hepatic acini.” Each of these units is hexagonal in shape and has a portal triad at each corner around its periphery (13). Each portal triad includes a branch of the hepatic portal vein, a branch of the hepatic artery, and a branch of the biliary tree. At the center of each lobule is the centrilobular vein (Figure 1). Sinusoids (i.e., capillaries with large permeable pores) connect the portal triad veins and arteries to the centrilobular vein (13). Oxygenated blood flows from the hepatic artery into the sinusoids where it mixes with nutrient-rich blood delivered from the small intestine via the hepatic portal vein (Figure 2). The highly porous sinusoidal endothelium ensures that the oxygen- and nutrient-rich blood can highly perfuse the surrounding hepatocytes as it flows to the centrilobular vein and enters the systemic circulation. As the oxygen is utilized by these hepatocytes, it creates an [O_2_] gradient across the lobule (i.e., from the periphery to the center). This enables each lobule to be split into 3 zones based on the O_2_ concentration, with hepatocytes in each zone having zone-specific functionality (Figure 1) (14).

Hepatocytes are the parenchymal cells of the liver and account for 60% of all liver cells (7). Differential expression of transporters between the basolateral and apical membranes ensures they are suitably equipped to uptake and efflux compounds from and into the blood and eliminate those destined for biliary excretion (e.g., toxic metabolites, bile acids, and bilirubin—the potentially toxic by-product of haem degradation) (15, 16). Alongside biliary excretion, hepatocytes have several other important functions, including (1) detoxification of ammonia via the urea cycle, (2) energy storage in the form of glycogen, (3) production of energy yielding precursors (i.e., pyruvate, glucose, and acetyl-CoA) via glycogenolysis, the Cori cycle, amino acid metabolism, beta-oxidation, and gluconeogenesis, (4) synthesis of cholesterol, bile acids, and ketone bodies, (5) lipoprotein metabolism, and (6) synthesis and secretion of serum proteins (e.g., albumin and coagulation factors). However, as stated, the function of a specific hepatocyte will be dependent on its location in the lobule (i.e., its zone). For example, zone 1/periportal hepatocytes tend to be specialized toward cholesterol synthesis, fatty acid oxidation, dehydrogenase-mediated oxygenation, sulfonation, and gluconeogenesis; whereas, zone 3 are specialized toward bile acid synthesis, glutamine synthesis, lipogenesis, CYP450-mediated oxidation, glucuronidation, and glutathione conjugation (14, 17).

Non-parenchymal cells (NPCs) make up the remaining 40% of cells in the liver and include the sinusoidal endothelial cells, stellate cells, Kupffer cells, and cholangiocytes (7). Sinusoidal endothelial cells form the leaky capillaries throughout the liver, enabling high perfusion of hepatocytes with plasma. Dispersed throughout the sinusoidal endothelium, Kupffer cells reside as liver macrophages that continually survey the circulating blood for foreign pathogens/ particles. They can act as antigen presenting cells to activate the adaptive immune response and play an important role in the inflammatory response, removing compromised cells and secreting pro-inflammatory cytokines (18, 19). They also remove damaged red blood cells and those at the end of their life, breaking down haem into bilirubin, ready for its removal by hepatocytes (18). Stellate cells represent 5–8% of cells in the liver and are located in the space of Disse, between the sinusoidal endothelial cells and hepatocytes (Figure 2). They store lipid-soluble vitamins such as vitamin A and play a part in regulating blood flow. Importantly, during liver injury and inflammation, stellate cells can reduce their lipid content and transdifferentiate into α-smooth muscle actin (α-SMA) positive myofibroblast-like cells, which deposit large amounts of extra cellular matrix (ECM) and secrete pro-inflammatory cytokines, creating a pro-inflammatory and pro-fibrogenic environment (20). Although part of the wound-healing process, excessive remodeling of the liver ECM and loss of parenchymal liver cells can lead to the development of scar tissue, liver fibrosis, regenerative nodules, which, as the liver loses its functional capacity, can progress to liver cirrhosis and liver failure (21, 22). Cholangiocytes are epithelial cells that line the biliary duct and facilitate biliary excretion by modifying the bile pH, its composition, volume, and permitting the re-uptake of components from the bile (e.g., glucose, amino acids, and ions) (23, 24).

## Metabolism and Drug-Induced Liver Injury

**Figure 3:**
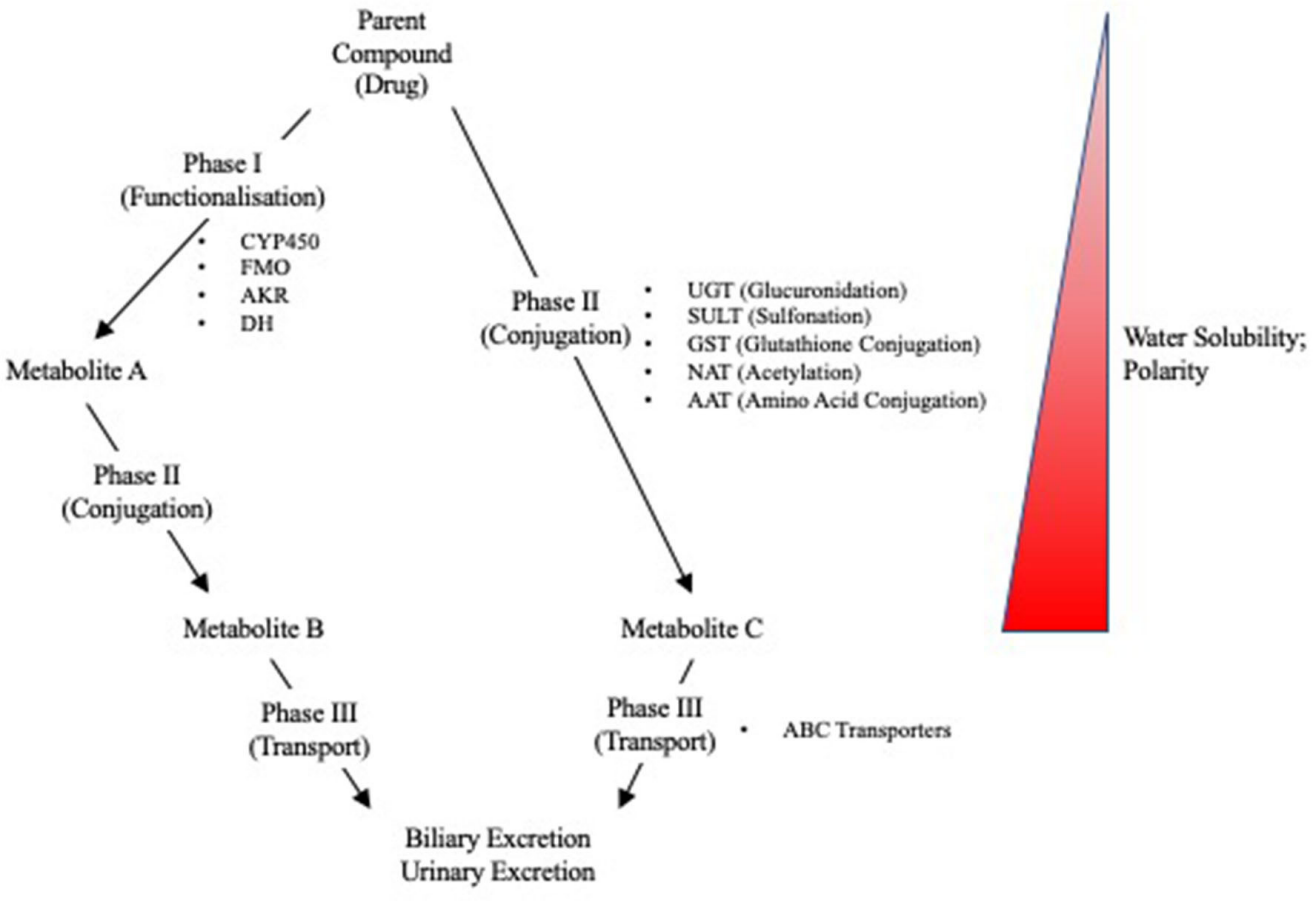
Overview of Phase I, II, and III reactions for xenobiotic metabolism and excretion. CYP450, cytochrome P450; FMO, flavin monooxygenases; AKR, aldo-keto reductases; DH, dehydrogenases; UGT, UDP-glucuronyl transferase; SULT, sulfotransferase; GST, glutathione-S-transferase; NAT, N-acetyl transferase; AAT, amino acid transferase; ABC, ATP-binding cassette.

Due to the liver's role in first pass metabolism, detoxification, and elimination of compounds, it can be exposed to high concentrations of drugs and their metabolites. It is, therefore, especially vulnerable to drug-induced injuries associated with genetic variation in drug metabolizing enzymes and transporters (DMETs) or perturbation of their function (e.g., induction or inhibition). Metabolism and detoxification of xenobiotics typically consists of three phases (i.e., phase I–III), and the expression of many of these proteins is regulated by several nuclear receptors. Functionalization of xenobiotics is carried out in phase I metabolism and involves the addition or exposure of functional groups to permit their conjugation to polar compounds in phase II metabolism. The resulting conjugates are generally more water-soluble, charged at physiological pH, and thus better substrates for phase III transporters, enabling hepatic excretion into the bile (Figure 3). CYP450 enzymes play an important role in phase I metabolism of lipophilic compounds, with CYP1, CYP2, and CYP3 families metabolizing 70 to 80% of approved drugs (25). Although CYP450-mediated metabolism is an important pathway for detoxifying many drugs and their toxic metabolites, it can also generate reactive metabolites that can react with and disrupt cellular macromolecules (e.g., lipid membranes and proteins), resulting in hepatocyte toxicity (26). In fact, the DILI risk for a drug is considerably higher if it is a CYP450 substrate (27). Flavin-containing monooxygenases (FMOs), although contributing to a much lesser extent than CYP450 enzymes, also contribute to the phase I metabolism of many drugs, with FMO3 being the most abundant isoform in the adult human liver (28, 29). Other families involved in phase I metabolism include aldo-keto reductases, monoamine oxygenases, esterases, epoxide hydrolases, and alcohol/aldehyde dehydrogenases (30). Phase II reactions involve the conjugation of xenobiotics with glucuronic acid, amino acids (i.e., glycine or taurine), a sulfonate group, an acetyl group, a methyl group, or glutathione via glucuronidation, amino acid conjugation, sulfonation, N-acetylation, methylation, and glutathione conjugation, respectively (31). Proteins involved in phase III metabolism include members of the ATP-binding cassette (ABC) transporter family such as MRP2 (ABCC2), BSEP (ABCB11), and P-glycoprotein (ABCB1). Drugs that inhibit these transporters, especially BSEP, have an increased risk for causing DILI (32). Importantly, many of these DMETs are also highly polymorphic, with some individuals having gene variants that cause loss-of function (poor metabolizers) or gain-of function (extensive metabolizers). This can cause over production or inefficient removal of a drug or its metabolite, resulting in its accumulation to cytotoxic concentrations that lead to DILI. There are now a number of examples of how genetic variation in DMETs or perturbation of their function (e.g., induction or inhibition of DMETs by drugs, foods, or disease states) can predispose an individual to DILI, including isoniazid and diclofenac (Table 1).

**Table 1:** Examples of drugs that can cause DILI, the drug metabolizing enzymes and transporters implicated in their toxicity, and the proposed mechanism(s).

**Drug (Class)**	**Toxicity**	**Implicated DMETs**	**Mechanism**	**References**
Acetaminophen (Analgesic)	Hepatotoxicity	CYP2E1	↑ NAPQI → ↑ Protein Adducts	(33)
Isoniazid (Anti-tuberculosis)	Hepatotoxicity	CYP2E1, NAT2, GSTM1	Mechanism unclear and controversial (↑ reactive metabolite(s) → ↑ protein adducts?)	(34–38)
Diclofenac (Analgesic)	Hepatotoxicity	UGT2B7, CYP2C8, ABCC2	↓ Glucuronidation → ↑ quinonimines → ↑ cell stress	(39, 40)
Ticlopidine (Anti-platelet)	Hepatotoxicity	CYP2B6 and HLA	Mechanism unclear (immune mechanism?)	(41)
Tolcapone (Anti-Parkinson)	Hepatotoxicity	UGT1A	Mechanism unclear (↓ glucuronidation → ↑ tolcapone → ↑ mitochondrial toxicity?)	(42)
Troglitazone (Anti-diabetic)	Hepatotoxicity/Cholestasis	CYP3A4?, SULT1A3?, UGT?	Mechanisms unclear and controversial ↑ TGZ-sulfate → ↑ BSEP inhibition → ↑ intracellular bile acids → Cholestasis → Mitochondrial toxicity → apoptosis ↑ TGZ → direct mitochondrial toxicity ↑TGZ-quinone → ↑ reactive intermediates (e.g., TGZ-epoxide, open-ringed metabolite, or superoxide anion radical) → ↑ protein adducts, oxidative stress, mitochondrial toxicity	(43–45)
Fialuridine (Antiviral)	Hepatotoxicity	ENT1	Fialuridine is transported into mitochondria by ENT1 → metabolized to its triphosphate derivative → inhibits polymerase-gamma → mitochondrial depletion → lactic acidosis and liver failure	(46, 47)
Bosentan (Anti-hypertensive)	Cholestatic	BSEP	BSEP inhibition → ↑ intracellular bile acids → cholestasis	(48, 49)

The metabolic heterogeneity for hepatocytes across each liver lobule can also result in zone-specific toxicities. For example, drug-induced toxicities that are associated with CYP450 activation may become more apparent at the centrilobular region of the liver (i.e., zone 3) at lower concentrations, due to higher CYP450 activity in these hepatocytes. Likewise, drugs activated by dehydrogenases may become more apparent at periportal hepatocytes (i.e., zone 1). Classic examples of this include the hepatotoxic compounds carbon tetrachloride and allyl alcohol, which cause centrilobular and periportal toxicity, respectively (50). Hence, certain toxicities may only be detectable at physiologically relevant concentrations in models that can sufficiently recapitulate a specific zone of the liver.

After identifying the gene polymorphisms that predispose an individual to developing hepatotoxicity, patients can be stratified into low- and high-risk groups for developing DILI based on their genetics. This allows a personalized medicine approach to prescribing, with high-risk patients being given a modified dose or, if possible, a safer alternative. Unfortunately, the exact gene variant responsible for a person developing DILI is often unknown and may be difficult to identify due to the relatively rare incidence of DILI, gene-gene interactions, and environment-gene interactions.

The complexity of the liver severely complicates the development of a single *in-vitro* model capable of modeling all the desired features. To be able to detect DILI with a metabolic basis, *in-vitro* models must be metabolically competent (i.e., expressing the aforementioned DMETs to sufficient concentrations), display hepatocyte functionality, and, ideally, be patient specific. It is also clear that drug-induced toxicities may be mediated by, or involve, multiple cell types (e.g., non-parenchymal cells such as fibroblasts, stellate cells, Kupffer cells), and thus may require co-culture model systems to detect (7). Moreover, the ideal model system to study DILI should have good sensitivity and specificity to detect DILI-positive compounds, indicating that the adverse outcome pathway is present within that system.

## Establishing *In vitro* Liver Models Using Human-Based Cells

The complexity of *in-vitro* liver models that utilize human-based cells can range from simpler monoculture models, which include a single cell type, to those with two or more cell types (i.e., cocultures and organoids). Importantly, the model should be able to recapitulate some functional aspect of the cells or organ that you are trying to model. Being the parenchymal cell of the liver, hepatocytes are a critical component for establishing an appropriate liver model, and in the case of metabolism-dependent DILI, they need to be metabolically competent for hepatocyte-specific DMETs. Currently, human-derived hepatocytes that are used for establishing these models are derived from multiple sources. These include primary human hepatocytes (PHHs), which are derived from liver patients that have undergone liver resection or biopsy (51, 52); cancer cell lines, which have been isolated from liver tumors (53–55); immortalized cell lines, which are hepatocytes that have undergone genetic modification to prevent cell death and promote survival (56, 57), and hepatocyte-like cells (HLCs), which are derived from pluripotent or adult stem cells (58–61). Each cell type has its own advantages and disadvantages (discussed throughout), but when they are grown *in vitro*, they will often be phenotypically different from their *in-vivo* counterparts for which you are trying to make conclusions (62–66). To try to overcome these phenotypic differences, a number of techniques have been used to try to recapitulate the hepatocyte's native environment *in vitro*, and evidence is gathering to suggest that growing cells in 3D (67–74) or exposing them to shear stresses and flow (75–77) can promote a more physiologically relevant model to study DILI, based on hepatocyte function and metabolic capabilities.

Other cell types used in combination with hepatocytes to establish *in-vitro* liver models include the non-parenchymal cell types found in the liver (i.e., Kupffer cells, stellate cells, liver sinusoidal endothelial cells (LSECs), and cholangiocytes), with the aim of recapitulating the liver's multi-cellular environment and paracrine signaling. Similar to PHHs, NPCs can be primary human cells derived from liver resection/biopsies (e.g., NPC liver fractions or isolated Kupffer cells) (32, 68, 78–81). Cancer/ immortalized cell lines can also be used to represent the liver-specific NPCs (82). For example, THP1 cells (a human acute monocytic leukemia cell line) and hTERT-HSCs (an immortalized human stellate cell line) have been used as Kupffer cell and stellate cell surrogates, respectively (82). Endothelial cells and mesenchymal stem cells are also being used in combination with hepatocytes to try to emulate the endodermal, endothelial, and mesenchymal interactions that occur during organogenesis and liver bud formation (83), with human umbilical vein endothelial cells (HUVECs) being used (83, 84) or endothelial cells derived from pluripotent stem cells (72, 85). Importantly, data indicates that the addition of these different cell types can promote hepatocyte maturation and metabolic competence *in vitro* (32, 68, 71, 72, 78–80, 82–86) and, therefore, may be important for detecting some mechanisms of DILI.

## 3D Cultures

**Figure 4:**
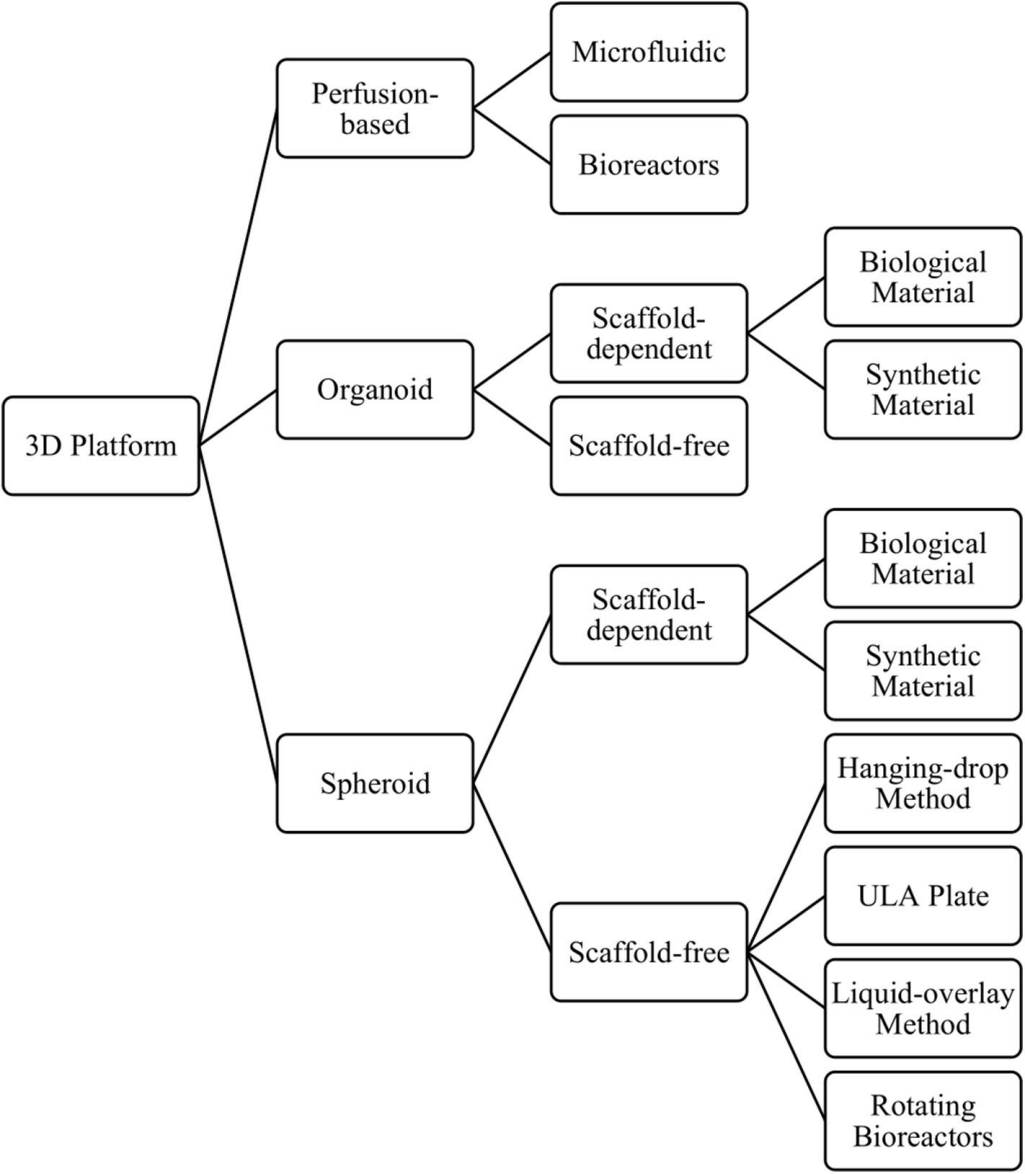
Overview of the different 3D models and methods used to produce them.

There is growing evidence indicating that culturing cells in 3D can promote a more *in vivo*-like phenotype than the same cell type cultured in 2D, when considering hepatocyte function and expression of DMETs (69, 71, 87–90). Consequently, they show greater sensitivity to detect toxicity for known hepatotoxic compounds, when compared to 2D cultures (12, 69, 81, 87). Another major benefit of growing cells in 3D is the ability to culture cells for longer, enabling longer and repeat dosing regimens that more closely resemble those seen in the clinic, which in turn allows the detection of previously undetectable chronic toxicities (e.g., fialuridine) (47, 68, 71). Three-dimensional *in-vitro* liver models include spheroids, organoids, and perfusion-based platforms, which can be further classified by their size, the presence and composition of a scaffold, and the technique used to form the cell aggregates (Figure 4), discussed below.

### Organoids or Spheroids?

**Figure 5:**
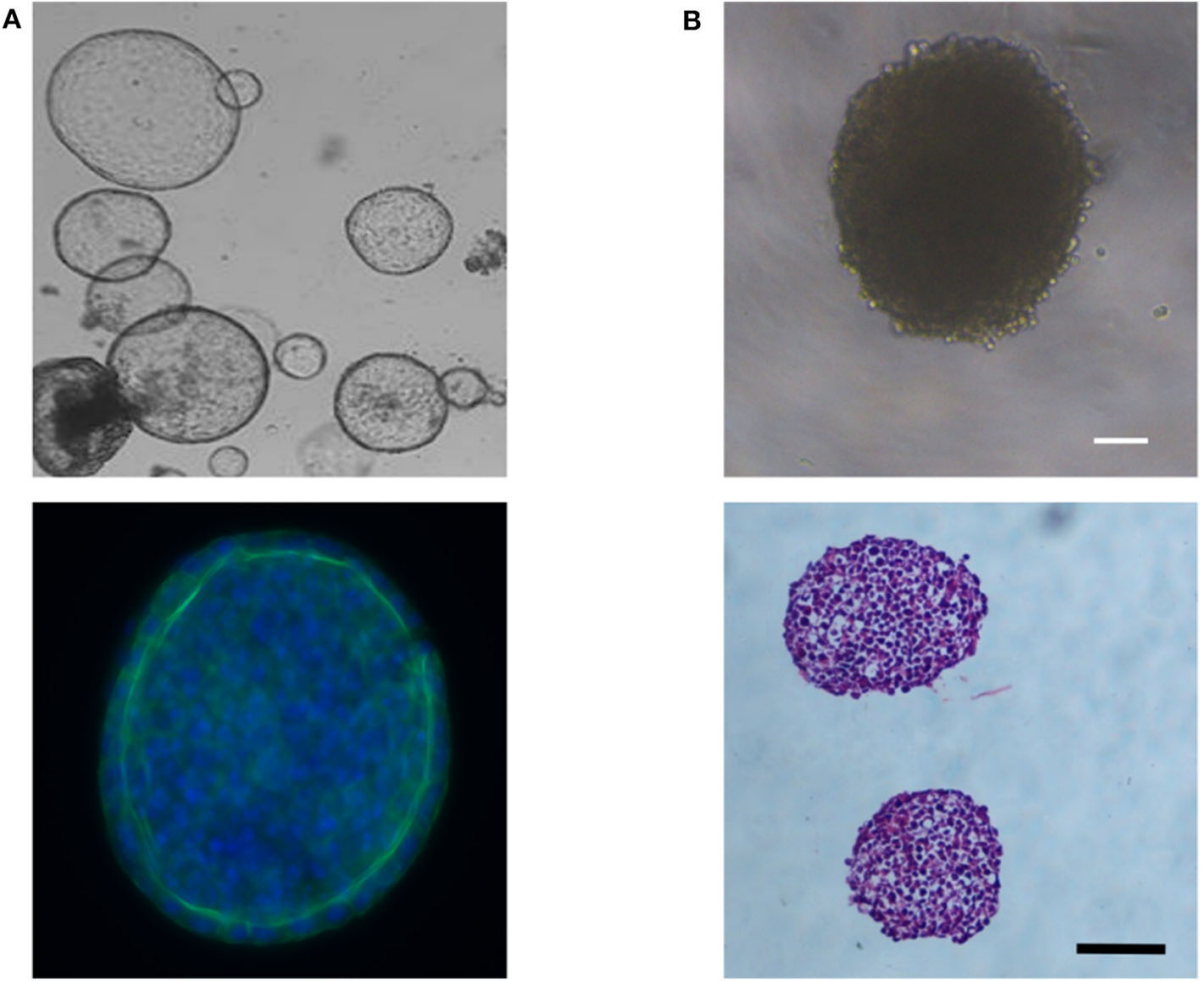
**(A)** Phase contrast image showing the cyst-like morphology of liver organoids derived from bipotent EPCAM^+^ biliary cells (Top image) and immunofluorescent image showing a layer of hepatocytes around the organoid's periphery; blue (Hoechst) = nuclei; green (Anti-E-cadherin antibody) = E-cadherin, the epithelial marker for hepatocytes (Bottom image). **(B)** Phase contrast image showing the dense morphology or HepG2 spheroids (Top image) and Haematoxylin and Eosin stained cross-sections of two HepG2 spheroids (Bottom image). Scale bars are 100 μm.

Liver spheroids and organoids have the benefit that cells are grown as 3D structures, allowing the development of cell-cell interactions, similar to that seen in their *in-vivo* environment. Data indicates that this can help promote a more mature hepatocyte phenotype *in vitro* (74, 91). Although the meaning of the term organoid has evolved over time, it can typically be defined as a self-organizing 3D structure, which includes multiple cell types, and is derived from pluripotent stem cells (83, 85) or tissue-resident cells isolated from biopsies (92–94), and can recapitulate in some way the structure and function of a specific organ (95, 96). Organoids form in response to chemical cues and cell-cell interactions that follow the developmental time course for that organ (96, 97). The morphology of liver organoids can vary from cyst-like structures (Figure 5A) (92) to more dense structures (83, 94) depending on the initial cell type(s) used to form the organoid. In contrast, spheroids are aggregates of cells that typically form a solid spheroidal structure (Figure 5B), which can include one or more cell types and can recapitulate some functional aspect(s) of an organ. Organoids and spheroids that contain multiple cell types (i.e., cocultures) are typically considered to have more physiological relevance, due to being able to recapitulate the multi-cellular environment that occurs *in-vivo*, and data suggests this can promote a more mature hepatocyte phenotype (see Co-cultures and Organoids sections). Certain toxicities may also involve multiple cell types, and, therefore, may only become detectable in these heterogenous models. However, due to the increase in complexity and the contribution of multiple cell types to the parameters of interest, data analysis and interpretation can become more challenging. Monoculture spheroids, in contrast, have the advantage of being able to do single cell-type analysis without the use of techniques such as fluorescence-activated cell sorting (FACS) to separate cells into their constitutive parts, enabling non-confounding metabolic analysis.

### Self-Assembly or Scaffold Systems?

Although some cell types can form 3D structures without any scaffold or matrix present, others may need a solid support system in order to form. As the name suggests, scaffold-dependent methods to produce 3D cultures relies upon a synthetic or biological scaffold or matrix to grow cells in a 3D format. The use of such scaffolds can also help cells establish a 3D structure with a particular architectural arrangement, which can now be achieved with extraordinary precision using bioprinting techniques (98). Biological scaffolds can also help to recapitulate the cell's *in-vivo* environment by including interactions between cells and the extracellular matrix (ECM) and mimicking the mechanical forces inflicted on cells (e.g., matrix stiffness), which can play an important role in cellular signaling, cellular behavior, and cell fate (99, 100). When using scaffolds, however, it is important to understand the effect that the scaffold has on cellular phenotype, any toxicity associated with the use of synthetic materials, and the extent of binding between drugs/proteins/compounds and the scaffold. Scaffolds can vary from decellularized liver tissue (101) to semi-synthetic (102) or fully synthetic constructs (103). Fully synthetic scaffolds have the advantage of being more reproducible, whereas decellularized liver tissue can retain the original architecture of the *in-vivo* liver. In contrast, scaffold-free methods rely upon self-assembly of cells into a 3D structure that permits the formation of cell-cell interactions. The methods used to produce such cultures include the hanging drop method (104), liquid overlay technique (69), the use of ultra-low adhesion plates (71), or rotating bioreactors (105) (Figure 4).

### Longevity

The ability to culture and dose cells for longer is an important advantage of 3D spheroids, when compared to 2D monolayers; however, the longevity of these model systems is limited by the development of a hypoxic and necrotic core or loss of hepatocyte function. Development of a necrotic core, however, is size-dependent and, therefore, typically associated with 3D spheroids formed with proliferating cells, which become larger over time, limiting the diffusion of oxygen into its core. This can be avoided by using mature, non-proliferating cells. The diameter at which hypoxia becomes apparent is often quoted to be 400 μm (106), but this will vary across cell types and should be determined experimentally, as different cell types will have different oxygen consumption rates, some of which have been modeled *in silico* (107, 108). Several studies indicate that 3D spheroids can be maintained for at least 1 month, following optimisation for cell seeding number (67–69), with reports of iPSC-derived hepatocyte-like cell spheroids being functional for up to 365 days (109). The number of proliferating cells within spheroids also tends to decrease over time, with proliferating cells becoming localized to the periphery when using proliferating cell types, which may be due to limited diffusion of oxygen and nutrients into deeper regions of the spheroid and inefficient removal of waste products, which together cause cells to become quiescent (10). Data also indicates that cells at the spheroid's core may develop a mesenchymal phenotype (i.e., undergo epithelial-mesenchymal transition), although they are yet to be fully characterized (109).

### Dosing Window

Selecting the correct time point for dosing or carrying out experiments may also be important for a specific toxicological study, as data indicates that expression of albumin and a number of important DMETs increases over time for HepG2 spheroids, with some only beginning to increase after 7 or 14 days in culture (67). The addition of non-parenchymal cells to cultures can also promote the expression of hepatocyte markers in 3D cultures and promote organoid maturation (84). Although both paracrine signaling and cell-cell interactions promote these changes, the 3D organization of organoids seems to be dependent on cell-cell interactions between parenchymal cells and non-parenchymal cells (84).

## Immortalized/Cancer Cell Lines

Many of the current *in-vitro* liver models involve the use of immortalized or cancer-derived cell lines, and although these models are robust, reproducible, and relatively cheap, the cells used are often phenotypically different from *in-vivo* hepatocytes (62). These cell lines tend to have abnormal chromosome numbers, chromosome abnormalities, and oncogenic mutations that promote survival (110–114), and thus may be more suitable to model cancer than drug-induced liver injury. Their ability to detect drug-induced liver injury is also limited, as many fail to express important enzymes involved in xenobiotic metabolism (115) and they lack the genetic variability to study patient-specific adverse drug reactions. Protein expression also varies considerably across cell lines (62, 116), as does their response to drugs (117), so choosing the most appropriate cell line is no trivial process and will be dependent on the question(s) of interest. Commonly used cancer-derived cell lines include HepG2, HepG2/C3A, Huh7, and HepaRG. Although phenotypically different from healthy *in-vivo* hepatocytes, studies indicate that growing these cells in 3D can promote a more mature phenotype than the same cells cultured as 2D monolayers or sandwich cultures, based on hepatocyte function and expression of DMETs (Table 2).

**Table 2:** Comparison of cancer-derived cell lines grown in 3D with those grown in 2D monolayers.

**Cell Type**	**Finding (3D vs. 2D)**	**Reference(s)**
C3A	Increased albumin secretion in 3D spheroids	(69, 118)
	Increased CYP2E1 protein expression in 3D spheroids	
	Increase in CYP1A2 and CYP3A4 activity in 3D spheroids	(118)
	Urea secretion and CYP3A4 protein expression comparable in 2D and 3D spheroids	
	Increased CYP1A2 protein expression in 3D spheroids	
	Increased urea secretion in 3D spheroid	(69)
	3D spheroids develop bile-canaliculi-like structures	
	3D spheroids have increased sensitivity to detect some hepatotoxins	
HepaRG	Increased urea secretion in 3D spheroids	(73)
	Higher CYP2B6 activity in spheroids	
	CYP3A4 and CYP1A2 activity comparable in 3D and 2D cultures after 7 days	
	Increased albumin secretion in 3D spheroids, which increases over time in culture	(104, 119)
	Increased ApoB secretion in 3D spheroids	(104)
	Albumin secretion comparable between 3D spheroids and 2D monolayer	(88)
	Sensitivity to APAP, ketoconazole and chlorpromazine toxicity was comparable in 2D monolayers and 3D spheroids	
	Increased CYP1A2, CYP2B6 and CYP3A4 activity in 3D spheroids and more inducible	(70, 88)
HepG2	3D spheroids have increased expression for genes involved in metabolism and synthesis, whereas 2D spheroids have increased expression for ECM, adhesion and proliferation genes	(105)
	Decreased albumin secretion in 3D spheroids	(120)
	GSTO1, GSTT1, and glutathione synthetase protein expression lower in 3D spheroids	(90)
	More variation in protein expression data for 3D spheroids than for 2D monolayers	
	Increased CYP1A expression, activity and/or induction in 3D spheroids	(105, 121–124)
	Higher expression of nuclear receptors in 3D	(67)
	Increased CYP2E1 expression in 3D spheroids but lower CYP2E1 activity	
	Higher UGT and SULT activity in 3D spheroids	
	3D spheroids develop bile-canaliculi-like structures	
	Increased activity for CYP2C9, 3A4 and 2D6 in 3D spheroids	
	Increased albumin secretion in 3D spheroids	(104, 105, 121, 123–125)
	Increased ApoB secretion in 3D spheroids	(104)
	CAR and PXR functional in 3D spheroids but not 2D monolayers	(126, 127)
Huh7	PXR functional in 3D spheroids but not 2D monolayers	(126)

### HepG2

HepG2 cells have been shown to maintain certain hepatic functions, including secretion of A1AT, albumin, α1-acid glycoprotein, AFP, transferrin, lipoprotein, complement proteins, fibrinogen and plasminogen (53). However, proteomic analysis indicates that they more closely resemble fetal hepatocytes than adult hepatocytes (64). Thanks to inducible CYP1A1, HepG2 cells have previously been used to detect mutagens activated by this enzyme (128, 129). Unfortunately, due to low or undetectable expression for a number of other enzymes involved in drug metabolism (i.e., CYP2E1, CYP3A4, CYP2C9, CYP2C19, CYP7A1, UGT1A, NAT2, and ABCB11), their ability to investigate DILI with a metabolic basis is limited (62, 128). There are, however, examples of transfected HepG2 cell lines that express different CYP450 enzymes, making them better able to detect metabolism-mediated hepatotoxicity (130–132).

Data indicates that culturing HepG2 cells in 3D can increase expression of a number of DMETs (67) and promote the correct localization and function of nuclear receptors, CAR (127) and PXR (126). Spheroids also have higher levels of albumin secretion, when compared to 2D monolayers (104, 105, 123–125), although there is some contradictory data (120). Albumin secretion may also be influenced by spheroid size, with larger spheroids showing less secretion (123). Whether this is due to diffusion limitations remains to be determined. Growing HepG2 as spheroids can also influence the regulation of CYP1A, with the aryl hydrocarbon receptor (AhR) only regulating CYP1A1 in spheroids, whereas it regulates both CYP1A1 and CYP1A2 in 2D monolayer cultures (122). The difference in regulation for CYP1A2 seems to involve PXR and may also be cell line specific (122).

### HepaRG

**Figure 6:**
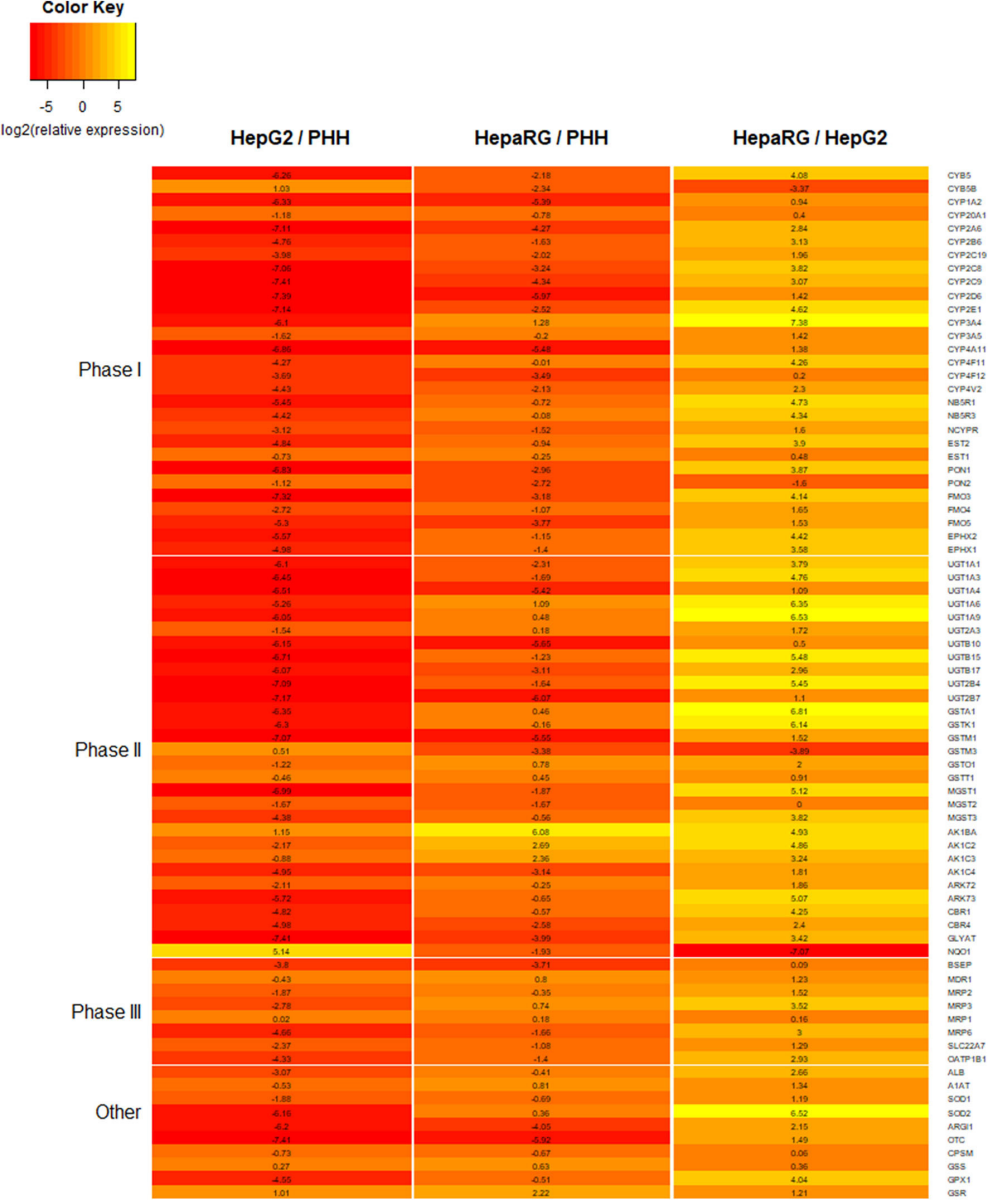
Heatmap comparing the expression for a number of proteins involved in xenobiotic metabolism and liver function. Expression data is for 2D monolayers: HepG2 relative to primary human hepatocytes (PHHs), HepaRG relative to PHHs, and HepaRG relative to HepG2. Data is reported as log2 of relative expression and sourced from a previously published study (116).

HepaRG is a bipotent progenitor cell line derived from a human hepatoma, which is capable of differentiating into a mixed population of hepatocyte- and cholangiocyte-like cells when treated with dimethyl sulfoxide (DMSO) (55, 133). When comparing different cell lines cultured as 2D monolayers, HepaRG show higher expression for a number of important DMETs, more closely resembling PHHs (Figure 6) (63, 89, 116). They also express inducible CYP450 enzymes, including CYP3A4, which, following induction, is comparable to basal expression and activity in freshly isolated PHH (63). In fact, HepaRG 2D monolayer cultures were shown to be superior to HepG2 3D cultures, when comparing DMET expression (134).

When HepaRG are cultured as 3D spheroids, the expression of a number of genes involved in gluconeogenesis, glycolysis, energetic lipid synthesis, bile acid metabolism, and lipoprotein metabolism increase in a time-dependent manner, and the expression of many of these was significantly higher than in 2D HepaRG cultures (104). A similar trend was seen for a number of phase I, II, and III proteins (88). However, contradictory data is seen for CYP3A4 activity, with some studies showing higher activity in 3D spheroids (70, 119) and another showing comparable activity (73). Comparable activity was seen for CYP1A2 with 2D monolayers and 3D spheroids (70, 73), although another study reported significantly higher CYP1A activity (i.e., using EROD assay) (119). CYP2B6 activity was shown to be higher for 3D spheroids (70, 73). HepaRG spheroids display inducible CYP450 enzymes in response to known pharmacological inducers, indicating functional nuclear receptors (i.e., PXR, CAR, and AhR) (70, 88, 104, 119). They also have glycogen synthesis and storage capabilities and develop bile-canaliculi-like structures (70), and have been shown to maintain viability for at least 21 (73) and 28 days (70). The superior DMET profile for HepaRG spheroids and the ability to repeat dose them over several day or weeks increases their sensitivity to detect hepatotoxicity for some known hepatotoxic drugs (70, 73, 119). They may also be able to better detect genotoxicity than 2D cultures (135). Due to being terminally differentiated cells, HepaRG spheroids are less likely to suffer issues with a hypoxic core, which can be problematic when using continually proliferating cell lines such as HepG2. However, due to the time required to grow and differentiate these cells, the high cost, and the loss of proliferation following differentiation, HepaRG are less amenable to high throughput analyses. The mixed population of biliary-like epithelial cells and hepatocytes following differentiation may help reveal mechanisms of toxicity involving both cell types, but in some cases could make the model more complex without adding much mechanistic benefit.

## Primary Human Hepatocytes

Due to being patient-specific and having sufficient expression of hepatocyte-specific genes upon isolation, primary human hepatocytes (PHHs) and liver tissue remain the “gold standard” for investigating drug-induced liver injury and patient-specific toxicities (8). Unfortunately, these can be of limited availability, require invasive procedures to source, and undergo rapid dedifferentiation when cultured *in vitro*, with reduced DMET expression occurring within 48 h of plating (66, 74, 136–138) and considerable transcriptomic changes detected as early as 30 min after plating (138). Data indicates that during this dedifferentiation process, cells acquire a regenerative-like phenotype with reduced activity for transcription factors involved in hepatocyte differentiation (e.g., HNF4 and HNF1a) and increased activity for those involved in stemness and proliferation (e.g., MYC), resembling that seen following partial hepatectomy (74). Importantly, unlike PHHs cultured as 2D monolayers, those cultured as 3D spheroids reacquire a phenotype indicative of a redifferentiation-like process (i.e., recovery of DMET expression—discussed below), following spheroid formation and re-establishment of cell-to-cell contacts, providing evidence for the importance of cell-cell interactions in PHH dedifferentiation and maintenance of the mature hepatocyte phenotype (74). Unlike the *in-vivo* regenerative process, however, the cells in 3D spheroids fail to proliferate to any appreciable level, which may be due to reduced Wnt/ beta-catenin signaling, leading to increased p53 activity and expression of PAI-1 (aka. Serpine1) (74), the latter of which has been associated with induction of replicative senescence (139). As well as loss of cell-to-cell contacts, several other factors associated with the isolation of PHHs have been implicated in the dedifferentiation process, including ischemia-reperfusion phenomenon, induction of an inflammatory response, and loss of cell-to-ECM interactions (136). Changes to the mechanical forces inflicted on PHHs when plated onto stiff substrates may also influence the hepatocyte phenotype (140).

When PHHs are cultured as 3D spheroids, the downregulation of ADME proteins is much less than in 2D monolayers (71). CYP1A2, CYP2C8, and CYP3A4 activity is also higher in PHH spheroids, with CYP2C9 and 2D6 activity being maintained for longer than in 2D sandwich cultures and monolayers (tested over 14 and 21 days) (71, 141). In fact, data indicates that PHH spheroids remain viable and metabolically competent, maintaining activity for CYP1A1, 2D6, and 3A4, for at least 35 days (68). CYP2C9 activity tends to increase over time, whereas CYP2C8 activity decreases over time (68, 141). Importantly, the activity for some CYP450 enzymes involved in drug metabolism is reported to be 10-fold to 1,000-fold higher than in 2D monolayers at day 21 of culture (141). There is still an initial drop in activity for a number of CYP450 enzymes in PHH spheroids, however, the loss of activity is at a much slower rate than in 2D monolayers and will often show some level of recovery over time (68, 71, 141). PHH spheroids also develop bile canaliculi-like networks and show a protein profile and ADME profile that more closely resembles the *in-vivo* liver than do 2D monolayers (68). Data suggests that albumin secretion is maintained over time in PHH spheroids cultured in a perfusion bioreactor, whereas urea secretion decreases over time until plateauing (142).

Due to their metabolic capabilities and the ability to culture them for longer, PHH spheroids have proven to be a useful model to study xenobiotic metabolism and identify human-specific metabolites (143). When incubated with several drugs, they were shown to be capable of generating the majority of metabolites detected *in vivo* for these drugs, indicating activity for a number of important CYP450 enzymes, UGTs, and SULTs. Unfortunately, the model was unable to detect NAPQI, the toxic metabolite of acetaminophen, which was likely due to low CYP1A2 and CYP2E1 activity, but this may have been detectable at higher doses (143), as the concentration of acetaminophen used (10 μM) in this study was much less than the IC50 (i.e., TC50) values reported for PHH spheroids, which tend to be above 320 μM (12). Nevertheless, when compared to spheroids established from other cell types, PHH spheroids are shown to be the most sensitive to acetaminophen toxicity (12). The improved metabolic activity for 3D spheroids compared to 2D monolayers may, at least in part, explain the greater sensitivity of 3D spheroids to detect known hepatotoxins at concentrations that are toxicologically relevant *in-vivo*, including for previously withdrawn drugs such as fialuridine and troglitazone (68, 71, 144). Thanks to expression of MRP2 and bile-acid-inducible BSEP and the ability to dose them for longer, PHH spheroids have also successfully been used to establish a model for detecting drugs with a cholestatic potential, which shows good sensitivity and specificity, following a 14 day repeat-dosing regimen (145). It is, however, worth noting that a HepaRG 3D spheroid model performed equally well (145). A PHH spheroid model cultured in defined conditions has also been described and shown to have relatively good sensitivity and excellent specificity (69 and 100%, respectively) for detecting DILI-positive compounds, in a model that uses an 80% viability threshold for identifying DILI-positive compounds and involves a repeat dosing regimen at 1x, 5x, and 20x Cmax (146). A separate study that used a margin of safety (i.e., IC50/Cmax) threshold for identifying DILI-positive compounds showed a sensitivity of 32, 55, and 61% when using a margin of safety threshold of 10, 30, and 50, respectively, which corresponded with specificities of 95, 87, and 79% (81).

## Stem-Cell-Derived Hepatocyte-Like Cells

Human embryonic stem cells (hESCs) and induced pluripotent stem cells (iPSCs) are pluripotent stem cells that are capable of self-renewal and generating all cell types in the adult human body (147). Hence, they provide an unlimited supply of patient-specific cells that can be used to generate patient-specific hepatocyte-like cells (HLCs) to study DILI (148, 149). The first reported protocol for the production of human hepatocyte-like cells involved the use of hESCs (150). These were cultured as spheroids at the hESC stage (i.e., producing an embryoid body), which were then treated with DMSO and sodium butyrate, and left to spontaneously differentiate into cells from all 3 germ layers, before selecting for HLCs (150). The use of hESCs, however, is a contentious area due to the ethical issues associated with the destruction of an embryo, which is required to isolate the hESCs (151). Their application for investigating the mechanistic details of patient-specific DILI is also limited, as models would be specific for the undeveloped embryo.

In 2006, Yamanaka et al. identified four factors, Oct3/4, Klf4, Sox2, and c-Myc, that were capable of reprogramming mouse fibroblasts into a pluripotent stem cell-like state (152). The following year, they reported a similar method to reprogram human fibroblasts to a pluripotent stem cell-like state that resembled hESCs (153). This would help overcome the ethical issues associated with hESCs and had the benefit of being patient-specific and readily available from easily isolatable cells. Since the original method described by (150), a number of different protocols have been described for differentiating pluripotent stem cells to HLCs with a greater efficiency and a phenotype that more closely resembles *in-vivo* hepatocytes. This includes the use of growth factors and small molecules (61, 154, 155), small molecules alone (59, 156), or transcription factors (157). These protocols aim to recapitulate the developmental process of hepatogenesis, directing the stem cells to differentiate into definitive endoderm, hepatoblasts, and finally hepatocyte-like cells.

Unfortunately, HLCs cultured as 2D monolayers still lack the maturity of *in-vivo* hepatocytes, displaying a phenotype that more closely resembles fetal hepatocytes (65). Due to the benefits of culturing cell lines and PHH as spheroids, a number of studies have looked at whether growing HLCs as spheroids can promote a more mature phenotype. These spheroids have been cultured at the stem cell stage of differentiation (i.e., producing embryoid bodies) (109, 158–160) or during the hepatoblast/maturation stage of differentiation (72, 87, 91, 158, 161).

Data indicates that culturing iPSCs as spheroids can promote a more efficient differentiation to the definitive endoderm, when compared to 2D cultures (162). This also seems to translate to HLCs, with differentiated HLC spheroids showing higher expression for a number of hepatocyte markers, including several CYP450 enzymes and GSTA1, with lower expression of mesenchymal markers (160). A1AT secretion decreased over time in both 2D and 3D cultures, but the rate at which it decreased was slower in 3D. Albumin and urea secretion was comparable over the 18 day study, although it was statistically significantly higher by day 18 (160). In a separate study, it was shown that CYP3A4 activity could be maintained in HLC spheroids for 1 year using a standard maintenance medium, although its activity and induction tended to peak around day 60 (109). Although albumin levels increased over the 30 day differentiation period, it was still much less than in human liver tissue (more than 80-fold lower). Expression of AFP also increased over these 30 days and was more than 300 times higher than in human liver, indicating an immature/fetal phenotype is still present at day 30. Nevertheless, HLCs continued to mature over time, with AFP secretion decreasing to negligible levels between days 20 and 90, whereas albumin secretion increased (109). Interestingly, spheroids in this study developed a peripheral layer of HLCs with what appears to be a vimentin-positive mesenchymal core, although this is yet to be fully characterized (109). The spheroid structure also seems to show polarity, expressing the apical membrane transporter BSEP and basolateral membrane transporter MRP1, the latter being localized to the inner side of the peripheral layer of HLCs. The number of proliferating cells also decreases over time, presumably as stem cells and progenitor cells became terminally differentiated HLCs (109).

Culturing HLCs as spheroids following differentiation or during the maturation stage can also promote a more mature hepatocyte phenotype, when compared to 2D monolayers (72, 91, 158). For example, increased activity for CYP3A4, 1A2, and 2C9, increased expression of UGT1A1, 1A6, MRP2, and BSEP, and increased secretion of albumin and urea, at day 11 of culture, when compared to 2D cultures at the corresponding time point (72). Furthermore, data indicates that CYP3A4 activity is maintained for at least 75 days in 3D cultures, whereas it is limited to 45 days in 2D cultures due to prolific cell death (91). Initial seeding density may also influence the culture morphology and phenotype, as spheroids formed from low seeding densities display a cyst-like structure following differentiation, whereas those seeded with a high number of cells are much more dense (160, 163). Larger spheroids show greater differentiation efficiency (i.e., higher expression for mature hepatocyte markers and lower fetal / biliary markers), and, contrary to that seen with immortalized / cancer cell lines, secrete more albumin per cell than smaller spheroids (123, 163).

Although HLCs offer great potential for investigating patient-specific toxicities, culturing and differentiating stem cells to HLCs is no trivial process. Alongside the immature phenotype of HLCs, the process is also very costly and labor intensive. The benefit of HLC spheroids for the safety assessment of drugs is still debatable until a comprehensive evaluation of their predictability is completed, but they could be important for investigating patient-specific toxicities.

## Co-Cultures

Using the hepatocyte as a single cell type to model DILI is, of course, an over-simplification and many mechanisms of hepatotoxicity will have a non-parenchymal cell component and involve multiple cell types. It may, therefore, be necessary to include these cell types when studying DILI. Indeed, 3D co-culture models have been established entirely from immortalized / cancer-derived cell lines (i.e., HepaRG cells, hTERT-hepatic stellate cells, and THP-1 monocytes) (82, 118), entirely from primary human cells (e.g., PHHs, Kupffer cells, stellate cells and cholangiocytes) (32, 68, 78–81), and entirely from the same iPSC (i.e., an isogenic model) (85). The addition of Kupffer cells and stellate cells provides a convenient model to study DILI with an immune-mediated mechanism of toxicity (78), investigate the roles of these cell types in drug-induced toxicities (82) and evaluate the pro-inflammatory and pro-fibrotic potential of drugs (68, 80). Cocultures that include just PHHs and Kupffer cells have also been described and used to evaluate the influence of Kupffer cell activation on a drug's toxic potential, with data suggesting that they can exacerbate toxicity for some drugs and be protective for others (81). Importantly, primary cell co-cultures can remain viable for 35 days, have inducible CYP450 enzymes, and maintain a relatively stable proteomic profile between day 7 and day 35, thereby allowing for repeat dosing studies to investigate chronic toxicities (78, 79). Nevertheless, there was a considerable decrease in expression for a number of phase I–III proteins by day 7, when compared to freshly thawed mixtures of PHH and NPCs (79). Notably, SLC22A1, CYP2C8, and CYP2E1 expression decreased rapidly over time, being more than 10-fold less at day 35, whereas, MRP2, P-gp, and vimentin increased by day 7 and then remained stable. CYP1A1, 2B6, 2C9, and 2C19 activity decreased over the 35 days, with peaks in activity often detected about day 14 to day 21 (79). When compared to 2D PHH monolayers treated for 48 hours, PHH and NPC co-culture spheroids (aka. liver microtissues) treated for 14 days have greater sensitivity to detect DILI-positive compounds, with comparable specificity (32).

The addition of non-parenchymal cells does not, however, guarantee a better model system to study DILI, with data suggesting that NCPs may reduce the expression of some CYP450 enzymes (80). Similar findings were seen with co-cultures that contain HLCs and stellate cells and HLCs and cholangiocytes (72). These detrimental effects, however, could be due to a sample dilution effect, resulting from the addition of cells that do not express these enzymes and proliferate when activated, causing them to overgrow the HLCs and PHHs in the spheroid (72, 80). Contrary to these findings, co-culture spheroids that include HLCs and endothelial cells have higher urea secretion and CYP450 activity than spheroid cultures comprised of HLCs alone (72). Furthermore, evidence is gathering to indicate that the addition of mesenchymal stem cells and endothelial cells can help improve the maturity of hepatocytes *in vitro* (i.e., albumin, urea, CYP450 markers), promoting hepatocyte polarization and 3D self-organization into structures with endothelial-like networks (72, 83–85, 164). Data indicates that cell-cell interactions between these cell types and paracrine signaling from mesenchymal cells and endothelial cells (i.e., FGF2 and BMP4) is important for this 3D self-organization and maturation (83, 84). After 12 days in culture, albumin secretion from these tricultures was shown to be comparable to human adult hepatocytes, although the gene expression of a number of hepatocyte markers still fell short of those seen in adult human liver tissue, approaching levels in fetal liver tissue (83). 3D spheroid co-cultures containing MSCs and PHHs, which have been grown in perfusion-based bioreactors, have also shown increased activity for some phase I and II enzymes, including CYP3A4, when compared to their respective 3D monocultures (77).

Reduced Wnt and TGF-beta signaling may also be important for hepatocyte maturation in 3D cocultures (159, 165). Interestingly, the inclusion of isogenic endothelial cells, compared to a heterogenic primary endothelial cell line, seemed to be more important than including isogenic mesenchymal cells, for maturation of hepatocytes, based on gene expression for several hepatic DMETs and albumin and AFP secretion (165). However, this may not be the case when using different primary cell lines or iPSCs and, therefore, needs further investigation.

It is also worth noting, however, that the addition of NPCs to PHHs grown as 2D monolayers can also increase activity for a number of CYP450 enzymes, although this only becomes apparent after 8 to 9 days in culture and SULT activity rapidly decreases over this period (166). Interestingly, these PHH-NPC 2D cocultures displayed less sensitivity and comparable specificity, when compared to PHH 2D monocultures, although the specificity for the cocultures increased with repeat exposures over 7 and 14 days (167), again highlighting the importance of exposure time and culture duration on the cells phenotype and sensitivity to known hepatotoxins (167).

## Organoids

Organoids derive from stem cells that have undergone chemically induced organogenesis, resulting in a 3D heterogeneous collection of cells that can recreate the organ microanatomy, containing phenotypically similar cells. Liver organoids can derive from liver progenitor cells (i.e., adult stem cell-like cells found in the liver) or pluripotent stem cells (i.e., ESCs or iPSCs). Organoid derivation from liver tissue involves the isolation of EPCAM ^+^ liver progenitor cells (aka. ductal “oval” cells) from liver resections/biopsies. These are then embedded into a Matrigel matrix and cultured with a combination of growth factors (i.e., HGF, EGF, FGF, Wnt, TGFβ, and Rspo1), causing EPCAM ^+^ cells to become activated bipotent liver progenitor cells that self-organize into a 3D spherical structure (92). The resulting 3D spherical organoids have a single-layered EPCAM ^+^ epithelial compartment, resembling the hepatic ductal compartment. Blockade of ductal fate by Notch inhibition, a potent inducer of ductal morphogenesis (168, 169), in combination with the inhibition of TGF-β signaling, removal of Rspo1, and addition of dexamethasone and BMP, facilitates the differentiation of organoids to a hepatocyte fate (92). These hepatic organoids have higher expression levels for several hepatocyte markers [cytochromes, albumin, or α-1-antitrypsin (A1AT)] and improved hepatic function (i.e., albumin production, cytochrome activity and bile acid production *in vitro*) (92). Organoids have also been developed without selecting for EpCAM ^+^ cells, from fetal liver tissue (94) or directly from cryopreserved PHHs (93, 94). Whether this is due to a small population of EPCAM ^+^ progenitor cells that remain after PHH isolation or dedifferentiation of PHHs to bipotent progenitor cells remains unclear.

Adult stem cell (ASC) derived organoids (i.e., from EpCAM ^+^ liver cell isolation) represent an attractive platform to model DILI, as they possess metabolically active hepatocyte-like cells that retain the genotypic and some of the phenotypic signatures of the original tissue, enabling the development of patient-specific models *in vitro* (92). Self-organization into structures that contain a central lumen also permits the diffusion of gases, such as O_2_ and CO_2_, nutrients, and metabolites, which may help to prevent the formation of a hypoxic or necrotic core. The requirement of surgical resections of healthy and/ or diseased tissue, however, can make it difficult to source the ASCs and brings about complex ethical issues. The use of iPSCs to generate organoids/liver bud organoids may help overcome these issues and offers a potentially unlimited supply of patient-specific cells.

Similar to ASC-derived organoids, iPSC-derived hepatocyte-like cells can retain certain phenotypic features of the original donor tissue, enabling the development of disease models (170). The first example of a human liver bud organoid derived from iPSCs was described in 2013, and included iPSC-derived hepatocyte-like cells, human umbilical vein endothelial cells (HUVECs), and mesenchymal stem cells (83). Including an endothelial and mesenchymal component into the model has the advantage of being able to investigate toxicities that involve multiple cell types. However, the use of genetically different cells can limit the application of the model. For this reason, they went on to establish a liver bud organoid that contained hepatocyte-like cells, endothelial cells, and mesenchymal cells, all derived from the same iPSC clone (85).

When comparing organoids produced from fetal and adult hepatocytes, fetal-derived hepatic organoids had less CYP3A4 activity than adult-derived hepatic organoids and PHHs, and also secreted more AFP, indicating, as one might expect, a more immature phenotype (94). However, AFP secretion did decrease over time, as the spheroids matured. Secretion of A1AT was lower than PHHs for both adult and fetal-derived organoids, but albumin secretion was comparable across them all (94).

Although morphologically similar, transcriptomic analysis of ASC-derived and ESC-derived hepatic organoids indicates differential expression for a number of genes, with the former showing up-regulation for genes associated with Wnt signaling, and the latter showing up-regulation for genes involved in liver development (171). When compared to 2D monolayers, embryonic stem cell-derived organoids display a more mature phenotype, based on several hepatocyte markers. However, they still fail to show the maturity seen with PHHs. Data indicates that hepatic organoids produced from fetal hepatocytes, adult hepatocytes, and pluripotent stem cells are capable of glycogen metabolism, LDL metabolism, and develop bile canaliculi-like structures (94, 171, 172). The addition of human fetal liver mesenchymal cells, which resemble stellate-like cells, can also promote maturation of ESC-derived hepatic organoids further in a co-culture model that could recapitulate the fibrotic pathogenesis associated with high ethanol exposure and thus alcoholic liver disease (171). It is also possible to generate cholangiocyte organoids, using the appropriate conditions (171). Hepatobiliary organoids, which are derived from iPSCs and contain both hepatocyte-like and cholangiocyte-like cells, have also been described in the literature (173).

Organoids derived from human fetal liver tissue have been maintained for at least 11 months, but the expansion time may vary depending on the starting tissue (i.e., fetal liver tissue or adult primary hepatocytes) (94). Patient-derived tumor organoids are also capable of recapitulating the features of the original tumor *in vitro*, providing a model for identifying oncogenic mutations with prognostic value in liver cancer and identifying potential drug targets, enabling a personalized medicine approach and a patient-specific model for testing a drug's efficacy and toxicity (174, 175).

Although liver organoids from ASCs and iPSCs provide an attractive platform to model DILI, there are still some hurdles to overcome. They are relatively labor intensive (e.g., isolating oval cells, maintaining stem cells, differentiating, characterizing, etc.), very expensive to grow, and the efficiency of organoid production is often low. The use of slightly different growth conditions and media can also result in low reproducibility for human organoid data in the literature.

## Perfusion-Based Systems and Microfluidic Devices

Perfusion-based *in-vitro* models have several benefits; (1) they can recapitulate vascular perfusion in the human body, (2) shear stresses and tension forces inflicted on the cell membranes are included in the model, enabling mechano-transduction, (3) connecting different cell types or organs under flow allows the transport of metabolites, cytokines, and signaling molecules, thereby considering the influence of different cell types or organs when assessing drug-induced toxicities, (4) pharmacokinetic studies are possible with the correct setup, (5) open-loop circuits enable repeat dosing experiments, (6) flow systems with air-liquid interfaces provide a model system to study permeability and barrier function, and (7) concentration gradients for nutrients, signaling molecules, and O_2_ can be setup across the system, which may help to establish liver models capable of modeling liver zonation *in vitro*, resembling that seen in the *in-vivo* setting. The use of automated stirred-tank bioreactors also provides a method to promote spheroid formation, whilst offering precise control of the culture parameters, which may help to prolong culture duration, enabling repeat-dosing and chronic toxicity studies (77, 142).

Data indicates that the flow rate and thus the shear stresses inflicted on the cell membrane can influence the expression of CYP450 enzymes, with higher flow rates promoting greater CYP1A2 activity in 2D and the opposite trend being seen in 3D (76). Similar findings for the effect of flow on CYP1A2 was also seen at the mRNA level (75). They also showed increased expression of CYP3A4 mRNA when exposed to flow (75). Another study indicates that albumin secretion, urea secretion, and CYP1A2 activity is increased when HepG2/C3A cells are exposed to flow; however, this did not correlate with the CYP1A2 protein expression data (118). The addition of flow also caused reduced expression of CYP2E1 and had no effect on CYP3A4 activity (118). A separate study showed that the addition of flow to PHH cultures had no effect on the activity for six CYP450 enzymes, but did cause a non-statistically significant increase in UGT and SULT activity (166). Nevertheless, the addition of flow to PHH/NPC cocultures could promote higher CYP450 activity after 24 hour in culture, when compared to cocultures not exposed to flow, with CYP3A4 and CYP2D6 activity increasing and decreasing over time, respectively (166). Comparing flow-based studies, however, can often be made difficult due to the use of different cell types, different flow rates / shear stresses, and different flow-based platforms (e.g., vessels and materials, etc.). The coefficient of variation can also be very large for some DMETs with some flow systems, which can be problematic when trying to develop a reproducible model (75). Although perfusion-based systems provide a convenient platform to model a number of biological barriers (176, 177), the benefit for liver models remains to be determined. Their greatest potential may come from their ability to develop multi-organ platforms to investigate the effect of one cell type or organ on the toxicity of another, in particular how hepatic metabolism contributes to drug-induced toxicities at other organs. For example, a heart-liver flow-based system has previously been used to study the role of hepatic metabolism in drug-induced cardiotoxicity (178). Perfusion-based models may also be useful for promoting the generation of mature hepatocyte organoids or HLCs derived from stem cells, as mechano-transduction and chemical gradients play a key role in developmental processes (179).

There are still many challenges with perfusion-based systems that must be considered, including non-specific binding in tubing and plates, especially microfluidic platforms that use PDMS (180, 181). For example, drugs with a partition coefficient <295 (i.e., LogP < 2.47 show minimal absorption (<10%) with PDMS, whereas those with LogP > 2.62 show extensive absorption (>90%) (181). Extensive testing of the flow-based system is, therefore, essential for robust and reproducible research. Bubbles and evaporation can also be particularly problematic for microfluidic devices due to the narrow channel design and use of small volumes (180, 182, 183). For more details on microfluidic devices, the reader is directed to several comprehensive reviews (183–185).

## Discussion and Future Perspectives

*In vitro* liver models provide a valuable tool to evaluate a drug's toxic potential and investigate the mechanistic details of DILI. Conventionally, this has involved culturing primary human hepatocytes or immortalized/cancer-derived cell lines as 2D monolayers. However, the cells used in these models will often be phenotypically different from *in-vivo* hepatocytes (i.e., cancer-derived cell lines), or rapidly dedifferentiate, becoming metabolically incompetent, when cultured *in vitro* (i.e., PHH). This hinders their ability to investigate metabolism-dependent toxicities and limits their application for detecting DILI-positive compounds, particularly over chronic and repeat dose time courses that are more emulative of *in-vivo* manifestations of DILI.

There is now a large amount of evidence to indicate that the phenotype of these cells can become more comparable to *in-vivo* hepatocytes and the dedifferentiation process delayed, when culturing the same cells in 3D. This includes higher albumin and urea secretion, and higher expression and activity for a number of important phase I-III proteins involved in xenobiotic metabolism (Tables 2–4). These beneficial effects may be due, at least in part, to the development of extensive cell-to-cell interactions, which, along with changes to the cell's shape and the mechanical forces inflicted on cells (e.g., tension), can influence cell signaling and cell fate (74, 186, 187). The ability to run three-dimensional cultures for longer may also be important for producing a more mature phenotype, with some cell types only showing increased expression for some metabolic proteins after 7–14 days (67). This also permits repeat dosing regimens, enabling the study and detection of chronic drug-induced toxicities (47, 145). The extra dimension to 3D models, however, tends to make them less reproducible than the same cells cultured in 2D (90).

**Table 3:** Overview of findings for 3D PHH spheroids and comparisons with 2D PHH monolayers and sandwich cultures.

**Finding (3D vs. 2D)**	**Reference(s)**
Ammonia metabolism/urea secretion is higher in spheroids	(73)
Gene expression is higher and/or maintained for longer for a number of phase I proteins in spheroid cultures	(68, 141, 144)
Activity for CYP450 enzymes involved in xenobiotic metabolism is higher and /or maintained for longer in spheroids	(71, 73, 89, 141)
Protein expression for a number of phase I, II, and III proteins is maintained for longer in spheroids	(71)
CYP1A2, 2C9, 2C19, and 3A4 more inducible to known pharmacological inducers	(89)
Gene expression for phase II and III proteins, nuclear receptors, and hepatocyte markers is higher and/or maintained for longer in spheroids	(141, 144)
GSTP1 gene expression lower in spheroids	(141)
CYP4A11 and SLC27A5 expression lost more rapidly in spheroids	(71)
Lower protein expression for some basolateral transporters in spheroids	(71)
Proteomic and metabolite profile is more stable in 3D cultures over time	(71, 141)
Spheroid cultures have greater sensitivity to detect toxicity for known hepatotoxic compounds at toxicologically relevant concentrations	(71, 73, 81)
Spheroid cultures show greater recovery from the dedifferentiation process associated with *in-vitro* culture, when considering gene expression	(74)

**Table 4:** Overview of 3D liver models that utilize hepatocyte-like cells (HLCs) and their phenotypic characteristics.

**Finding (3D vs. 2D)**	**Reference(s)**
Higher CYP3A4 expression in spheroids	(87, 163)
Higher CYP2C9 expression in spheroids	(87)
CYP2C9 and 3A4 expression is more inducible in spheroids	(87)
Greater albumin secretion in spheroids	(163)
Expression of fetal/immature hepatocyte markers (e.g., AFP and CYP3A7) are lower in spheroids	(72, 91, 163)
Gene expression for some UGTs and canalicular transporters is higher in 3D cultures	(72, 91)
Higher CYP1A2, 2C9, and 3A4 activity after 11 days in spheroids	(72, 91)
2D and 3D cultures both capable of lipid and glycogen storage	(91)

Cell selection is an important consideration and should be based on the parameter under investigation. Immortalized and cancer cell lines have the advantage of being well-characterized, reproducible, and robust. When compared to PHHs, the current gold standard, they also do not require invasive procedures to source and offer a potentially unlimited supply of cells. However, due to them being continually proliferating cells, 3D cultures can develop a hypoxic and necrotic core, which may limit how long they can remain in culture, although the 3D format still shows extended longevity over their 2D counterparts. This issue becomes less of a problem when using HepaRG cells, which can be differentiated to a mixed population of non-proliferating, terminally differentiated hepatocyte-like cells and biliary epithelial-like cells, prior to spheroid formation, enabling spheroid size to remain stable over time. Furthermore, they have metabolic capabilities (i.e., expression and activity of phase I-III proteins) that are approaching those seen in PHHs, which also improves when cultured as spheroids. They, therefore, offer a suitable cell to study DILI, but the extended time requirement necessary for their differentiation can limit their high throughput capabilities. The lack of genetic variation in cell lines can also prevent the detection of patient-specific toxicities resulting from gene polymorphisms, although this can be investigated using genetic manipulation, offering a convenient test system to investigate the findings from Genome-wide association studies (GWAS).

When it comes to investigating patient-specific toxicities with a genetic basis, HLC spheroids and organoids may provide an invaluable tool. Unfortunately, similar to PHHs, the production of organoids from isolated liver progenitor cells (aka. oval cells) requires liver resections. Nevertheless, there are a number of examples of organoids/organoid-like structures developed from iPSC-derived HLCs. These can provide an unlimited supply of patient-specific hepatocytes to study DILI, from easily isolatable cells (i.e., peripheral blood mononuclear cells—PBMCs or fibroblasts). They also have better metabolic capabilities than many cancer-derived and immortalized cell lines. The immature phenotype of HLCs, however, limits their application, and although growing them in 3D can promote a more mature phenotype, they still lack the maturity and metabolic capabilities of freshly isolated PHHs.

The benefit of adding different cell types to 3D hepatocyte cultures seems to be dependent on the cell types added. Data suggests that co-cultures with endothelial cells and mesenchymal cells may be the most suitable combination to promote a mature hepatocyte phenotype. However, the benefit of each non-parenchymal cell (NPC) type may be dependent on the mechanistic details of interest, as a number of drug-induced toxicities will have an immune-mediated response, which could initiate hepatotoxicity or cause a secondary insult (i.e., in response to the secretion of pro-inflammatory cytokines). Nevertheless, having multiple cell types in a model (e.g., organoids and spheroids containing multiple cell types) can make the data more difficult to interpret. Selecting the correct ratio of cells in coculture models is also an important consideration, as data suggests that this can influence the hepatocyte phenotype (118).

Based on a number of parameters for hepatocyte function and maturation, three-dimensional culture systems have a number of advantages over 2D monolayers; however, many of the established assays and analytical techniques are for 2D methods. Imaging and high throughput techniques can be particularly challenging for 3D cultures. Hence, the rate at which the field of 3D biology can progress is dependent on the analytical techniques, reagents, and culture plates available for 3D platforms. Although these are becoming more widely available, they tend to be more expensive than the 2D monolayer equivalents. However, the use of commercially available ultra-low adhesion plates can help increase the reproducibility of data, and there are a number of commercially available assay kits, optimized for use with 3D cultures (e.g., more sensitive to account for fewer cells in 3D spheroids compared to 2D monolayers). Attempts are also being made to optimize suitable microscopy techniques to assess toxicity using 3D cultures (188), although, based on their data, it is unclear whether HLC spheroids provide any advantage over 2D HLC monolayers for detecting DILI-positive compounds (188). However, the method of assessment could have a significant effect on the outcome. Protocols for producing high resolution images of 3D cultures using fluorescence microscopy have also been described (189).

In the future, perfusion-based systems may play an important role in helping to reveal the mechanistic details for drug-induced toxicities involving multiple organs. However, these systems still require a large amount of optimisation to culture cells from different organs in the same system for an extended time. Identifying the optimal flow rates to achieve physiologically relevant hepatocyte function can also be challenging and can change depending on whether cells are cultured as spheroids or monolayers. The reproducibility of these systems is also yet to be determined. The development of new technologies (e.g., nano sensors) that are biologically safe and capable of measuring parameters throughout these systems and at the cell membrane (e.g., [O_2_], shear stresses, etc.) may help in characterizing these models and understanding how these parameters influence biological processes and hepatocyte function. Consequently, many of the current flow systems have to rely on computational modeling to achieve this (190).

The metabolic capabilities of different cell types and models are extensively covered in this review and are an important consideration when establishing models to investigate and detect metabolism-dependent toxicities. However, there have been relatively few studies that have comprehensively evaluated a model's predictive capabilities for detecting DILI-positive compounds, with most using very small test sets, and in some instances only having two or less DILI-negative compounds. Although discussed throughout this review, a comprehensive review of the predictive capabilities of different 3D spheroid models can be found in a separate review (12). At current, PHH spheroids and PHH coculture spheroids have been the most extensively evaluated spheroid systems for their predictive capabilities (32, 81, 146). Importantly, the quality of a model for detecting DILI-positive compounds is dependent on both its sensitivity and specificity. Moreover, a model that has high sensitivity and low specificity offers little practical application in drug discovery and development, potentially leading to the unnecessary attrition of drugs and testing on animals. Likewise, *in-vitro* models with high specificity and low sensitivity may be indicative of being mechanistically inadequate to detect the toxicity of interest.

Although there are now a number of methods that can increase the complexity of 2D *in-vitro* models, it is important to understand their limitations and remember that an increase in complexity does not always translate to a better model. Moreover, the ideal model should be able to answer the question(s) of interest, whilst also being as simple as possible.

## Author Contributions

CC, SL, CG, and PS conceived and designed the study. CC and SL drafted the manuscript, which was then edited by all authors. All authors gave final approval for publication.

## Conflict of Interest

The authors declare that the research was conducted in the absence of any commercial or financial relationships that could be construed as a potential conflict of interest.
